# Role of Acute HIV Infection in Driving HIV Transmission: Implications for HIV Treatment as Prevention

**DOI:** 10.1371/journal.pmed.1001803

**Published:** 2015-03-17

**Authors:** Laith J. Abu-Raddad

**Affiliations:** 1 Infectious Disease Epidemiology Group, Weill Cornell Medical College in Qatar, Cornell University, Doha, Qatar; 2 Department of Healthcare Policy and Research, Weill Cornell Medical College, Cornell University, New York, New York, United States of America; 3 Vaccine and Infectious Disease Division, Fred Hutchinson Cancer Research Center, Seattle, Washington, United States of America

**Linked Research Article**

This Perspective discusses the following new study published in *PLOS Medicine*:

Bellan SE, Dushoff J, Galvani AP, Meyers LA (2015) Reassessment of HIV-1 Acute Phase Infectivity: Accounting for Heterogeneity and Study Design with Simulated Cohorts. PLoS Med 12(3): e1001801. doi: 10.1371/journal.pmed.1001801

Using simulated cohorts that account for previously unmeasured bias, Steve Bellan and colleagues provide new estimates of the duration and relative infectivity of the HIV-1 acute phase based on data from the retrospective cohort of serodiscordant couples in Rakai, Uganda.

Not many issues have been debated in the HIV community more than the role of acute HIV infection in driving HIV transmission in sub-Saharan Africa (SSA) [1]. Acute infection refers broadly to the first stage of progression following onset of infection during which, for several weeks, HIV viral load peaks at high levels (up to 10^7^ virions/ml of plasma), before leveling off at the viral set point for several years [2]. The debate is well beyond being an academic debate; the impact of different HIV prevention strategies is contingent on the role of acute infection in the epidemic. A large role for acute infection may erode some of the utility of antiretroviral treatment as prevention (TasP), the most promising HIV prevention intervention in our time [3].

TasP is a costly investment in HIV prevention. For it to be impactful and cost-effective, HIV-infected persons must be diagnosed early in their infection and linked to care and treatment. Despite promising developments [4], it is difficult in practice in resource-poor settings to identify and treat newly infected persons within the short duration of acute infection. If acute infection drives a large fraction of HIV transmissions, TasP may not curtail transmission chains as desired. This brings a critical question: how much of HIV transmission in SSA is driven by infected persons in their acute infection? In a research article published this week in *PLOS Medicine*, Steve Bellan and colleagues provide profound insights towards addressing this question [5].

## Role of Acute Infection in the Epidemic

Mathematical modeling studies have attempted to answer this question, but their estimates have varied widely, from as little as 2%–3% to as high as 75% of transmissions [2]. Looking critically at these studies, the inconsistency in findings does not seem to be due to different model structures, so much as to variable assumptions in model parameterization, such as the infectivity of persons in acute infection, the duration of this stage of disease progression, and the sexual network of these individuals. The role of acute infection was found to be highest in epidemiological contexts of high sexual risk and early epidemic phase. Phylogenetic studies suggested similar findings [2,6].

## Infectivity during Acute Infection

The cornerstone dataset for determining acute infection infectivity is the seminal Rakai study, a study of HIV transmission within 235 retrospectively identified HIV-discordant couples in a Ugandan population-based cohort [7]. Different analyses on this dataset indicated that infectivity was an order of magnitude higher for acute infection than for the chronic stage [2,7]. Bellan and colleagues have critically reanalyzed this dataset. In essence they reconducted the study, but in silico. Their analysis was motivated by examination of the impact of one bias, that of heterogeneity in infectiousness. Recruitment of HIV serodiscordant couples can be biased towards those who are persistently serodiscordant, as a consequence of their lower infectiousness. The researchers found this bias to have a significant effect on inflating acute infectivity estimates. However, they happened upon a more subtle finding.

Bellan and colleagues discovered a critical bias in the retrospective design of the Rakai study: the exclusion of couples who were observed to be concordant negative, and then observed to be serodiscordant only once before being censored because of loss to follow-up, couple dissolution, or the end of study. This bias alone caused acute infectivity to be overestimated by a factor of two. Accounting for different sources of bias, the researchers found acute infectivity to be five-fold higher than chronic stage infectivity, an estimate that is only about 10% of often-quoted estimates for acute infectivity [1,2]. They also found that this revised estimate is consistent with the estimate derived using viral load trajectory. The latter had been discounted earlier based on suggestive biological grounds [1,2]. It seems, after all, that HIV plasma viral load predicts HIV infectivity during acute infection, just as it predicts HIV infectivity during chronic infection.

Based on these new estimates, and using a previously constructed model that assesses the role of acute infection in the epidemic [8], acute infection may not contribute today more than about 5% of transmissions in the established hyper-epidemics of SSA, and no more than 10% cumulatively since the start of these epidemics in Africa. These numbers are smaller than even some of the most conservative estimates of acute infection contribution [2]. Skeptics of a major contribution for acute infection appear to have prevailed in this debate [1].

## Implications for Treatment as Prevention

Using the earlier estimates for acute infectivity, HIV elimination—defined as <1 incident HIV infection per 1,000 person-years [1]—is predicted to be difficult to attain unless TasP coverage among infected persons (other than those in acute infection) is extremely high, well beyond what is feasible [3]. With Bellan and colleagues’ estimate, however, HIV elimination in the shorter term can theoretically be attained at a lower coverage. Based on extension of an earlier model [8], it seems possible to eliminate HIV in the hyper-epidemics of SSA by 2050 with a TasP coverage of about 90%. While this coverage might still not be feasible, the new estimate for infectivity implies larger and faster incidence reductions with TasP.

An important consequence of the new estimate is an enhancement in the cost-effectiveness of TasP, which strongly influences donor investment in prevention strategy. Although, based on a simple calculation, the new estimate implies no more than 5% higher cost-effectiveness for TasP, such a small increment may have still significant budgetary implications. Another consequence is that interventions targeting those in early infection may not be, in comparison, impactful or cost-effective for averting HIV transmission. Early infection interventions face cost and logistical challenges, and may not be feasible in SSA. The saturated and declining epidemics in SSA further suggest a minor role for acute infection. It might be time to focus on TasP as the best strategy to get these epidemics under control, at least for the time being.

## Moving Forward

Bellan and colleagues’ study has shaken our faith in a result taken for granted for a decade. Their simulations estimate that acute infectivity is probably no more than a few times higher than chronic infectivity, and may not have contributed a large fraction of transmissions in the hyper-epidemics of SSA. These findings affirm the role that TasP can play in controlling the epidemic.

Despite Bellan and colleagues’ insightful analysis, the debate may not yet settle. Population-based trials of TasP will report their findings within the next few years [9], and they may provide a decisive answer. Moreover, a small role for acute infection in the hyper-epidemics of Africa may not translate into a small role for acute infection in concentrated epidemics in key populations elsewhere, such as among men who have sex with men, people who inject drugs, and female sex workers and their clients [2,10,11]. As the SSA epidemics decline and HIV epidemiology approaches the concentrated epidemic pattern, the relative role of acute infection may rise. TasP may prove to be the intervention that controls the “general population” side of the epidemic, while other complimentary strategies will be needed to reach an AIDS-free generation.

Bellan and colleagues’ analysis provides a cautionary tale. It took a decade to realize that the estimates of acute infectivity were inflated, and this could have been avoided. Simulation studies provide a powerful tool to assess the influence of sources of unavoidable bias in epidemiological study design. Different lines of direct and indirect evidence can contribute to build credibility for key results. Mathematical modeling work, for example, has already suggested a higher infectivity for the chronic stage than has been found in cohort studies of serodiscordant couples [12]. While complex analytics may add burden to the costs and logistics of epidemiological studies in the short term, this investment can minimize costs and optimize scientific value in the long term.

## Abbreviations

SSA: sub-Saharan Africa
TasP: treatment as prevention
